# Supplementary material for: The Effects of Paddy Cultivation and Microbiota Members on Arsenic Accumulation in Rice Grain
Source: Foods. 2023 May 26;12(11):2155. doi: 10.3390/foods12112155 (PMC10252851; doi:10.3390/foods12112155)
Supplement: Supplementary file 1 [file foods-12-02155-s001.zip › foods-2354998-supplementary.pdf]

SUPPLEMENTARY INFORMATION

The Effects of Paddy Cultivation and Microbiota Members on Arsenic Accumulation in Rice Grain

Esra Ersoy Omeroglu[1,*], Asli Bayer[1] , Mert Sudagidan[2] ,Veli Cengiz Özalp[2] , Ihsan Yasa[1]

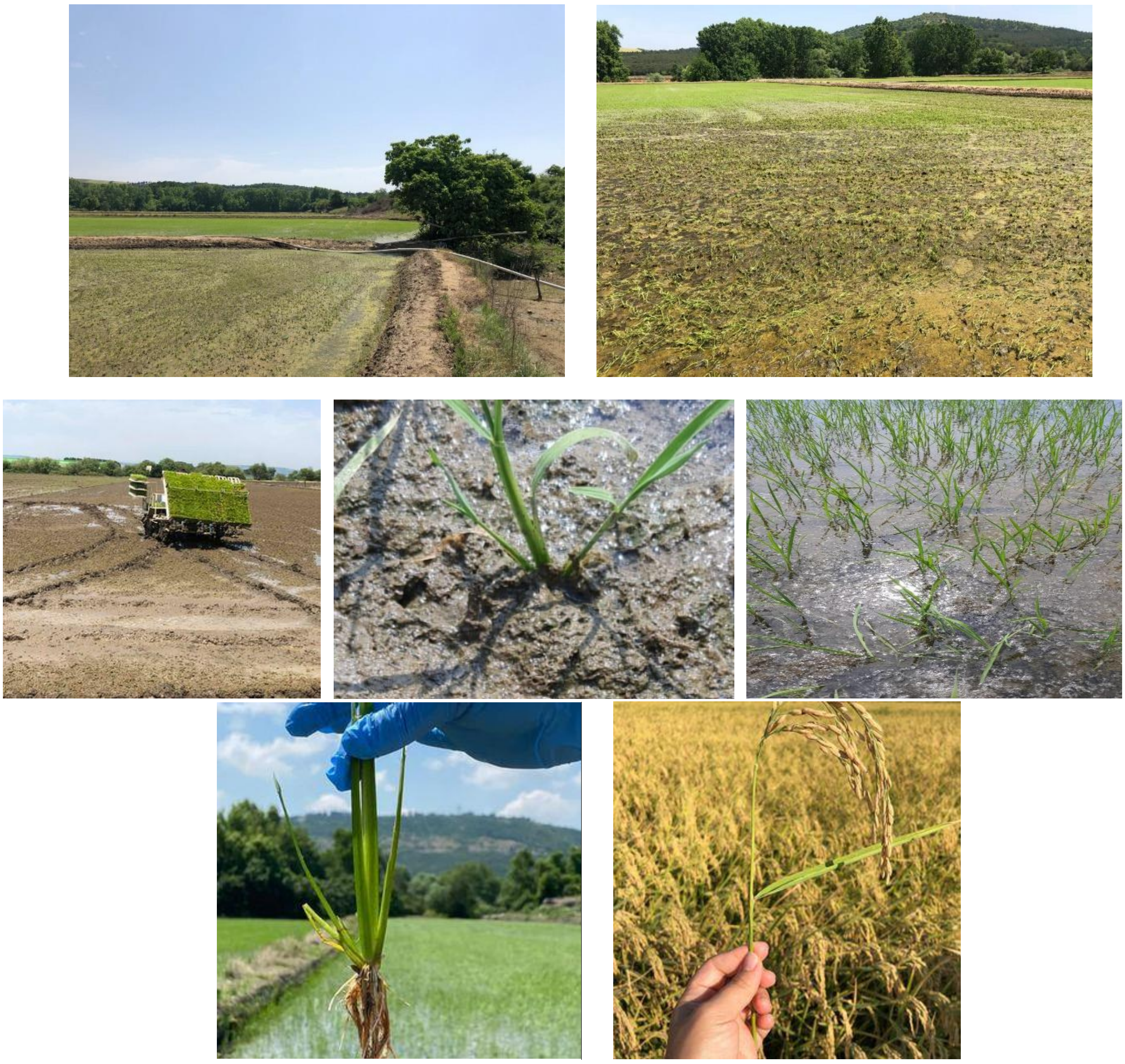

Figure S1. Various Images of Rice Sampling.

A

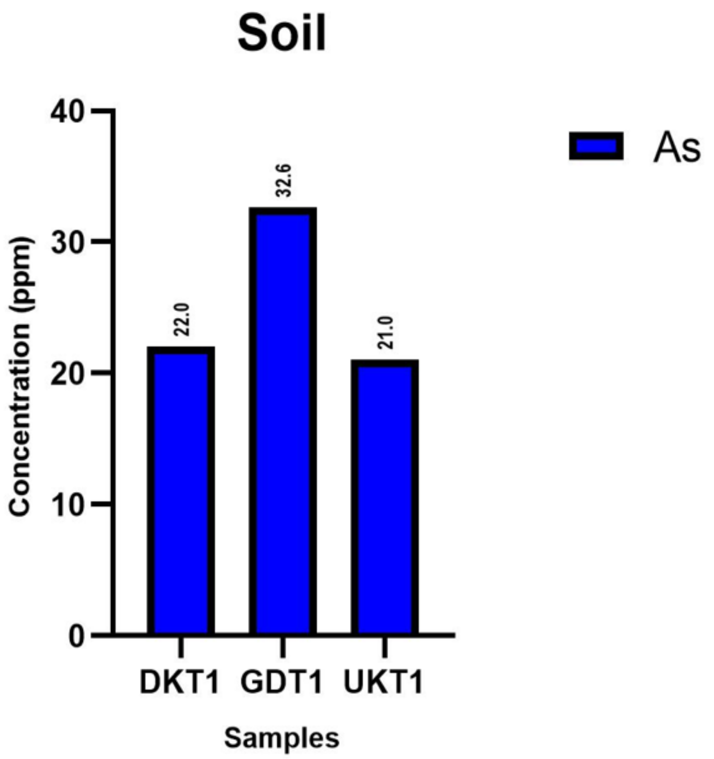

Table format: Grouped	Group A	Group B	Group C	Group D	Group E	Group F	Group G
	P	K	Mn	Fe	Cu	Zn	As
1 DKT1	64.834	3246.00	549.100	28666.9	24.4	76.0	22.0
2 GDT1	45.650	4331.30	867.900	38197.8	39.9	112.5	32.6
3 UKT1	73.436	2808.40	405.400	28190.9	21.5	68.5	21.0

B

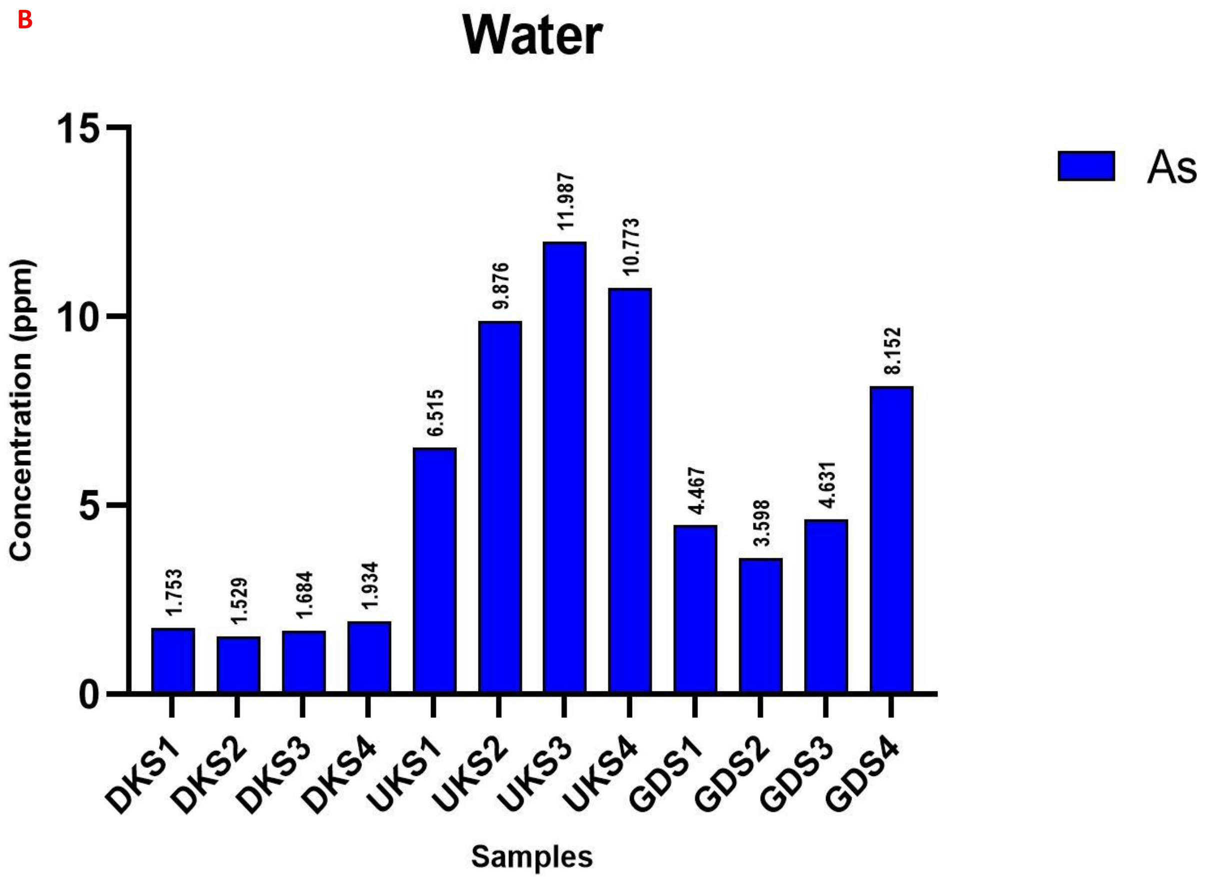

	Table format: Grouped	Group A	Group B	Group C	Group D	Group E	Group F	Group G
		P	K	Mn	Fe	Cu	Zn	As
1	DKS1	26.30452	3393.143	1.471	27.839	0.522	0.000	1.753
2	DKS2	7.85378	2848.286	3.036	0.740	0.020	3.356	1.529
3	DKS3	49.28000	2308.761	2.273	0.740	0.253	1.825	1.684
4	DKS4	93.00000	3991.365	7.227	0.740	0.473	19.008	1.934
5	UKS1	411.31270	7319.941	25.455	57.070	1.338	1.447	6.515
6	UKS2	635.20800	8356.629	1.237	11.702	2.216	11.401	9.876
7	UKS3	475.64000	7991.434	3.122	10.531	0.705	2.217	11.987
8	UKS4	828.00000	12443.950	5.484	0.740	0.775	14.949	10.773
9	GDS1	67.98924	2590.427	52.422	159.760	2.957	0.509	4.467
10	GDS2	33.73766	2520.335	0.314	2.152	0.671	2.349	3.598
11	GDS3	28.38000	2680.144	1.703	6.805	1.314	2.365	4.631
12	GDS4	46.00000	2760.579	0.040	0.740	0.853	16.313	8.152

c

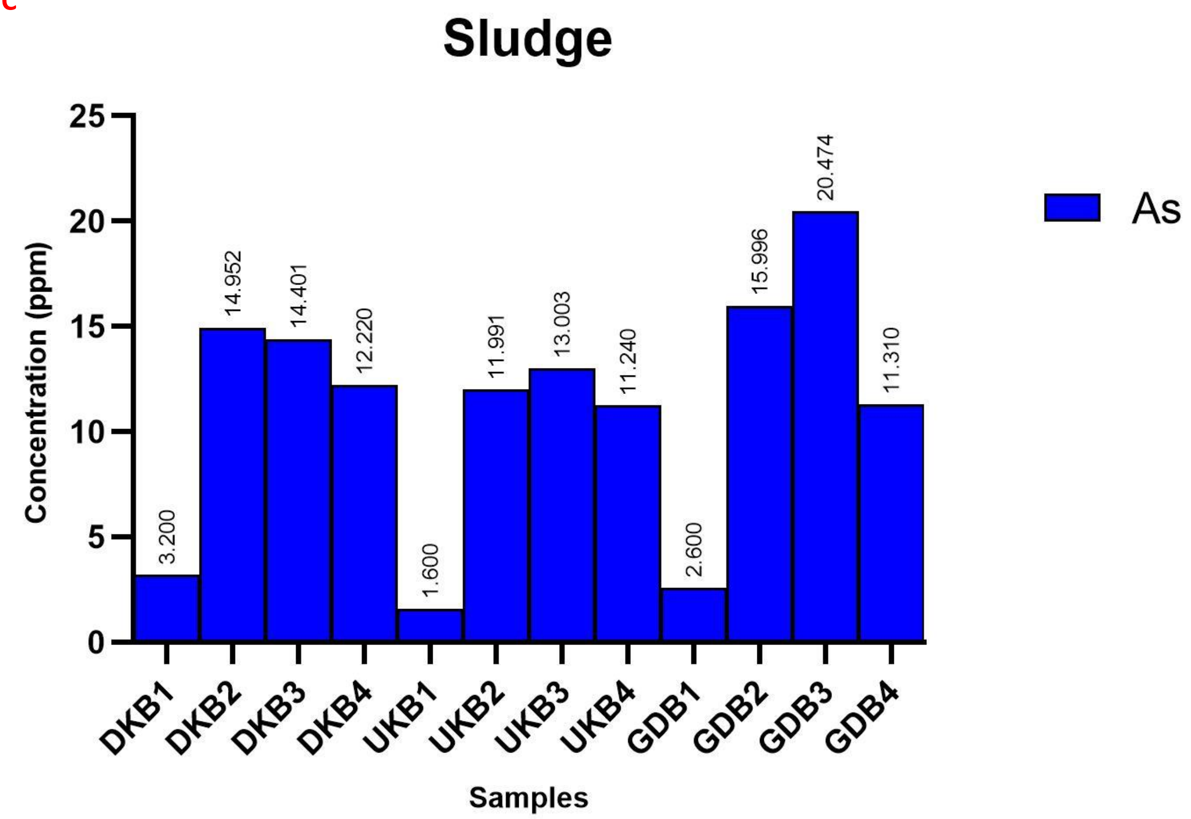

	Table format: Grouped	Group A	Group B	Group C	Group D	Group E	Group F	Group G
		P	K	Mn	Fe	Cu	Zn	As
1	DKB1	21.5820	475.300	83.600	3684.50	3.800	10.300	3.200
2	DKB2	347.6024	3127.697	336.854	20395.22	15.755	51.448	14.952
3	DKB3	438.2083	2301.892	336.494	18196.37	13.974	45.026	14.401
4	DKB4	214.4600	1278.580	143.980	8427.15	7.190	45.840	12.220
5	UKB1	17.7100	361.000	36.800	2197.20	2.100	6.000	1.600
6	UKB2	276.7959	2038.578	248.641	16528.71	12.054	37.302	11.991
7	UKB3	366.0094	1375.251	206.462	15015.74	11.475	34.944	13.003
8	UKB4	216.1900	1350.670	130.230	8850.39	6.520	38.020	11.240
9	GDB1	14.4540	519.600	72.400	3354.70	3.500	9.200	2.600
10	GDB2	271.9774	2230.848	378.413	21212.83	22.072	58.430	15.996
11	GDB3	402.6893	2230.617	383.521	23104.90	24.278	63.329	20.474
12	GDB4	139.6400	1170.630	151.210	6945.50	7.940	46.460	11.310

D

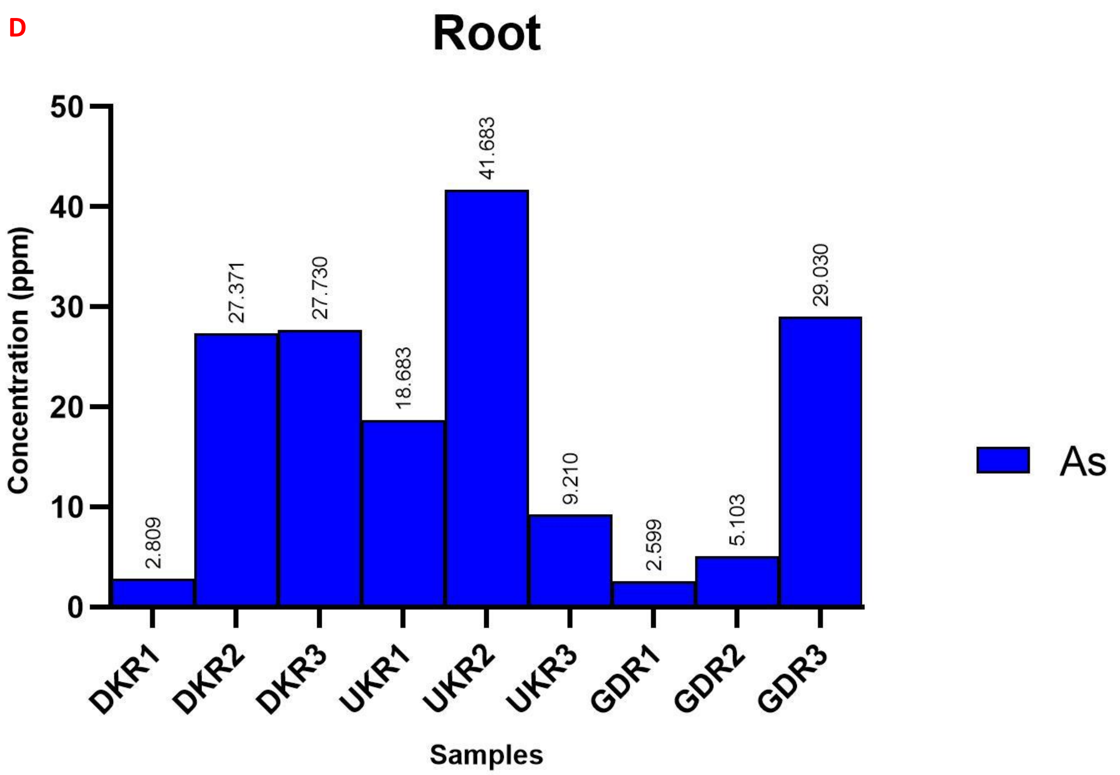

Table format: Grouped		Group A P	Group B K	Group C Mn	Group D Fe	Group E Cu	Group F Zn	Group G As
1	DKR1	546.83662	1613.993	83.158	1229.061	3.528	9.937	2.809
2	DKR2	372.46858	2509.630	112.677	3640.166	6.433	12.711	27.371
3	DKR3	177.32000	365.930	179.680	4957.900	4.690	54.420	27.730
4	UKR1	560.44208	1747.798	101.019	3407.661	7.224	12.525	18.683
5	UKR2	611.54324	1699.134	84.458	5811.640	2.745	9.483	41.683
6	UKR3	279.56000	618.480	113.460	2109.080	4.910	21.610	9.210
7	GDR1	779.36804	1499.451	74.557	1640.708	4.816	13.056	2.599
8	GDR2	219.35276	2314.840	63.274	1253.557	4.102	6.684	5.103
9	GDR3	119.70000	167.640	182.180	2389.630	2.400	12.450	29.030

E

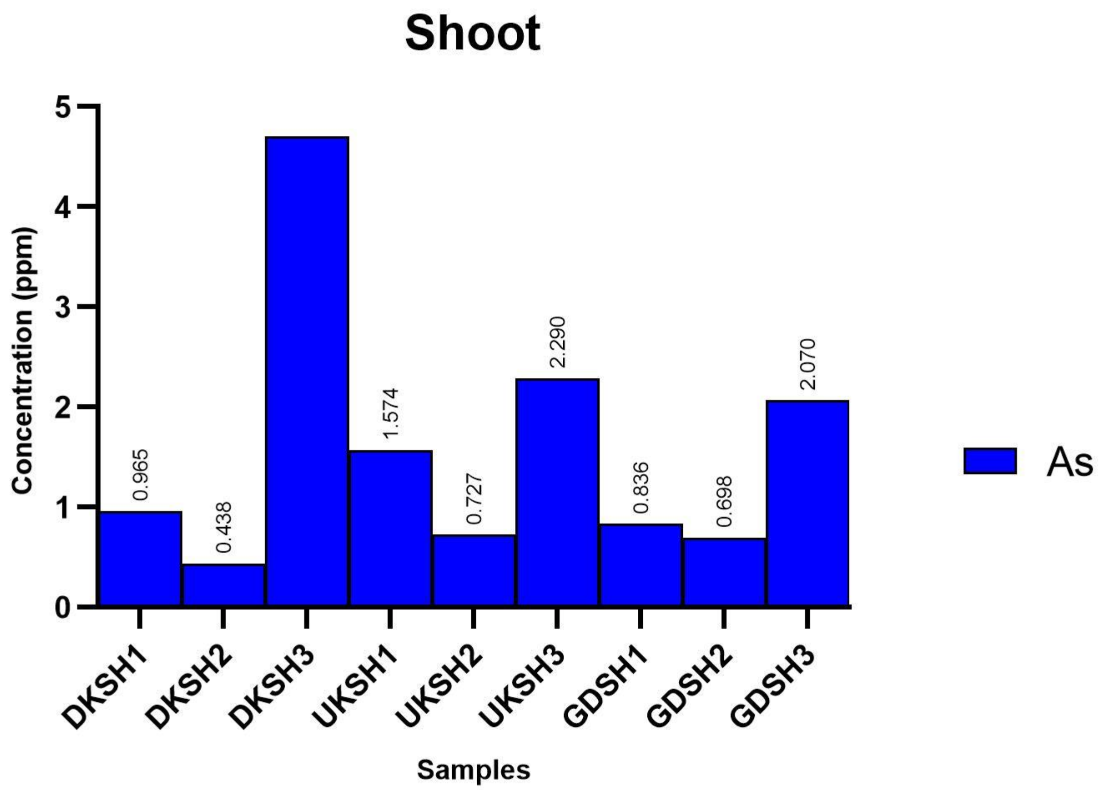

Table format: Grouped		Group A	Group B	Group C	Group D	Group E	Group F	Group G
		P	K	Mn	Fe	Cu	Zn	As
1	DKSH1	868.17676	7477.093	105.739	831.908	3.833	17.242	0.965
2	DKSH2	477.59052	4398.930	76.164	160.201	2.340	11.781	0.438
3	DKSH3	493.82000	8438.580	1209.730	324.240	1.670	18.800	4.700
4	UKSH1	749.70324	5692.137	133.261	1403.084	7.083	26.758	1.574
5	UKSH2	382.75600	2412.270	54.582	135.136	0.760	7.916	0.727
6	UKSH3	465.04000	4513.370	799.490	370.550	1.960	16.280	2.290
7	GDSH1	1391.29078	10096.011	125.296	662.468	5.938	25.443	0.836
8	GDSH2	665.00544	5330.514	126.919	211.562	2.674	12.656	0.698
9	GDSH3	179.80000	2608.340	747.710	459.940	6.020	15.600	2.070

F

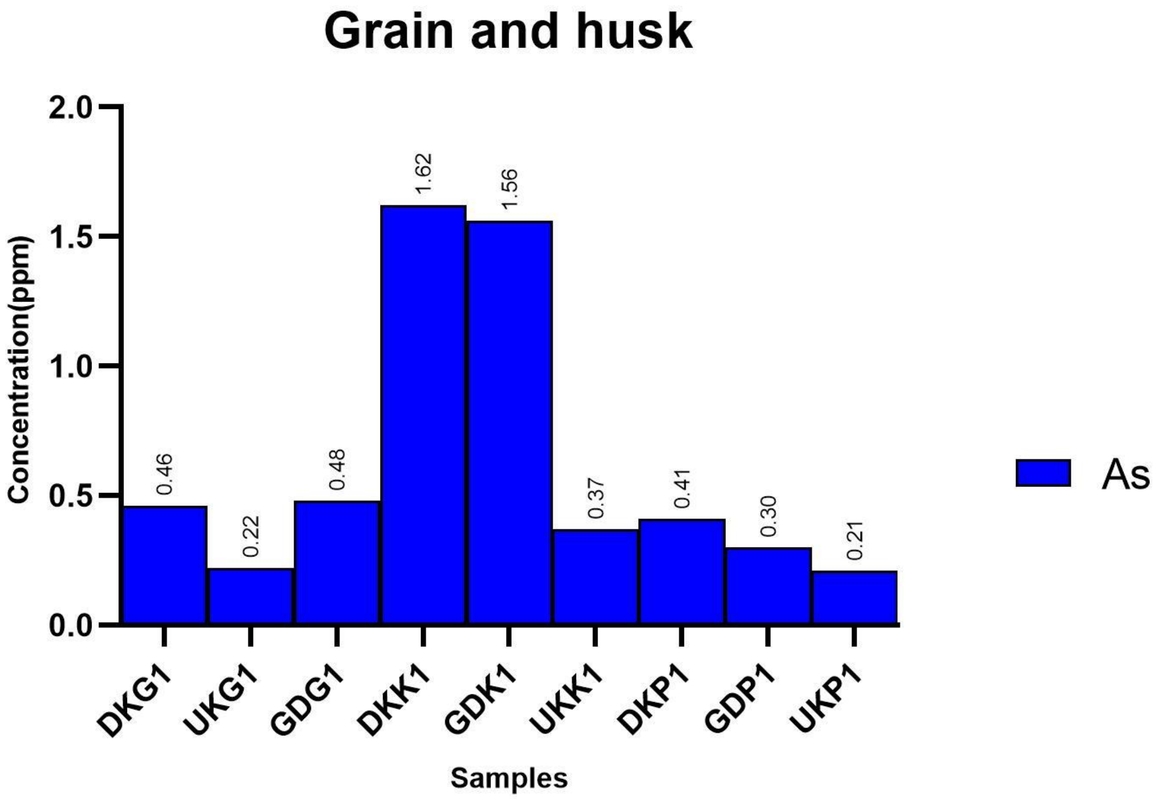

Table format: Grouped		Group A	Group B	Group C	Group D	Group E	Group F	Group G
		P	K	Mn	Fe	Cu	Zn	As
1	DKG1	2474.89	3273.70	113.09	71.32	3.01	18.77	0.46
2	UKG1	1994.49	2329.61	74.71	45.47	2.46	17.38	0.22
3	GDG1	2821.10	4551.39	148.00	65.32	3.96	24.33	0.48
4	DKK1	233.17	9439.42	200.06	129.54	1.58	13.42	1.62
5	GDK1	674.37	10355.64	270.65	169.32	2.52	16.14	1.56
6	UKK1	424.39	4846.94	268.82	78.15	1.75	15.29	0.37
7	DKP1	3125.30	4013.06	28.18	49.11	2.18	18.61	0.41
8	GDP1	3079.80	3428.45	28.14	54.63	3.15	18.87	0.30
9	UKP1	2865.89	3422.86	39.25	53.73	3.76	24.53	0.21

Figure S2. Graphics Related to Arsenic Amounts of Rice Agricultural Land Samples and Chemical Analysis Results. **A**: Soil, **B**: Water, **C**: Sludge, **D**: Root, **E**: Shoot, **F**: Grain and husk.

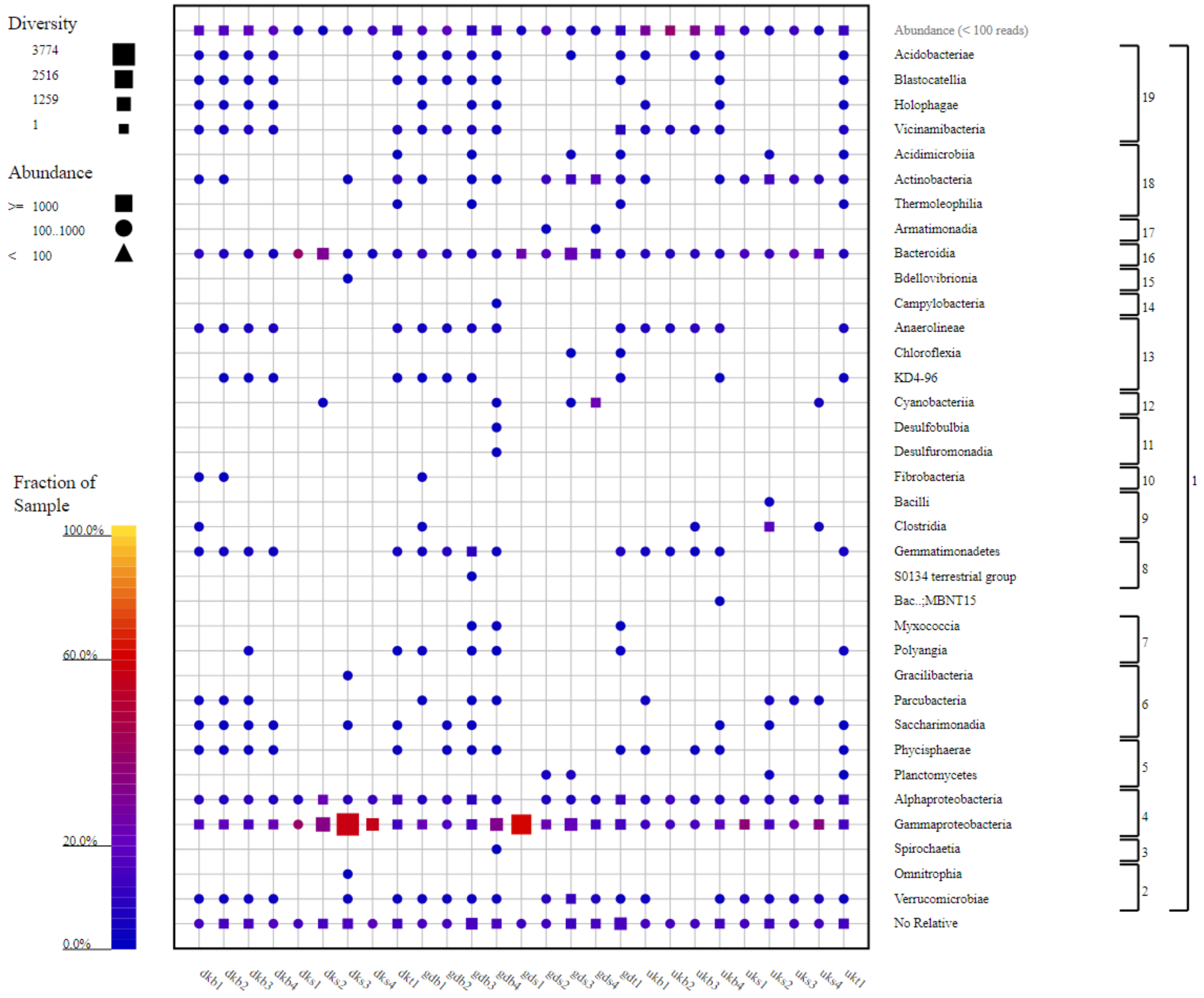

Figure S3. Classis-level taxonomic fingerprint of water, soil and sludge samples from rice farming (The figure was created using SILVA NGS 1.4 v138.1).

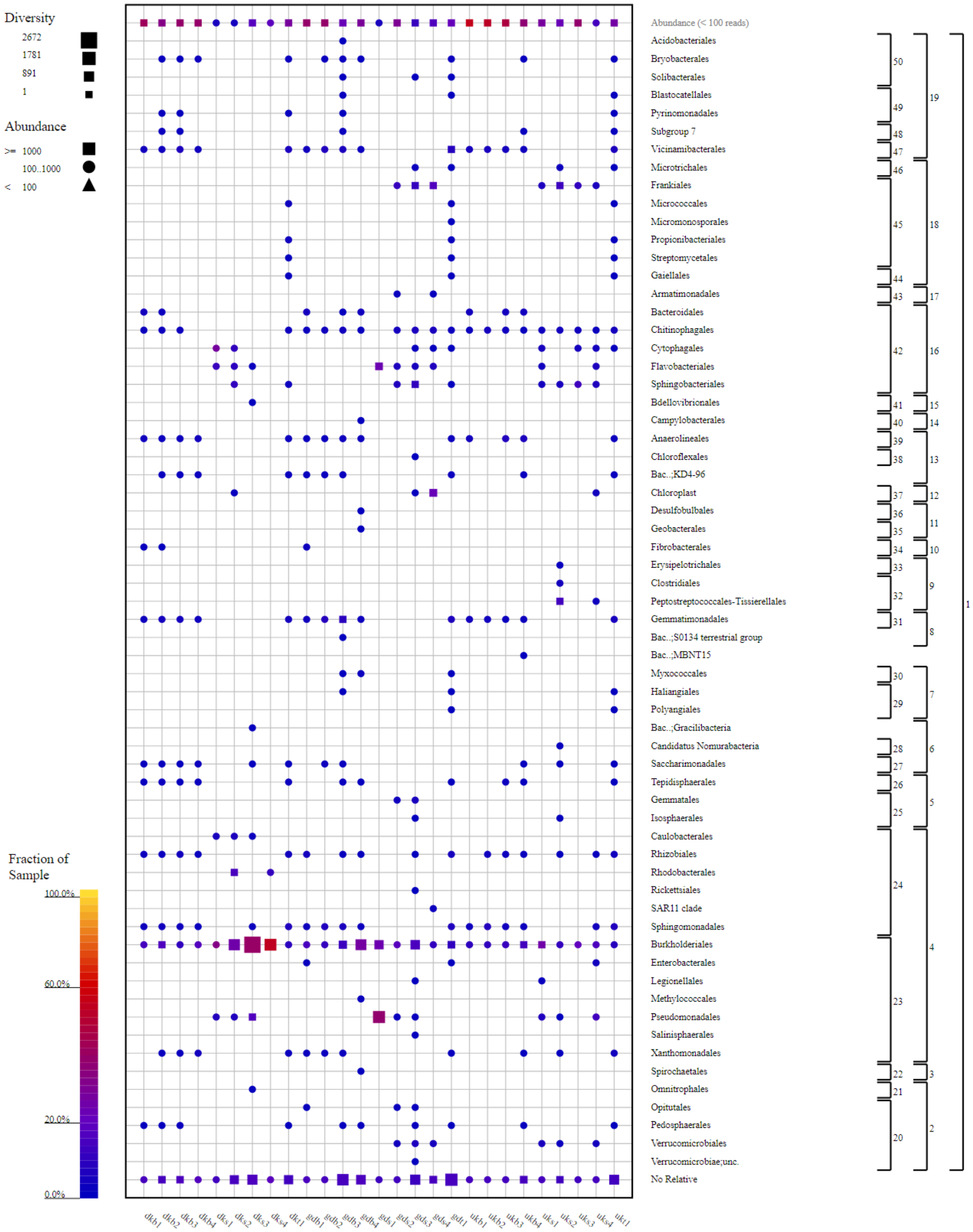

Figure S4. Order-level taxonomic fingerprint of water, soil and sludge samples from rice farming (The figure was created using SILVA NGS 1.4 v138.1).

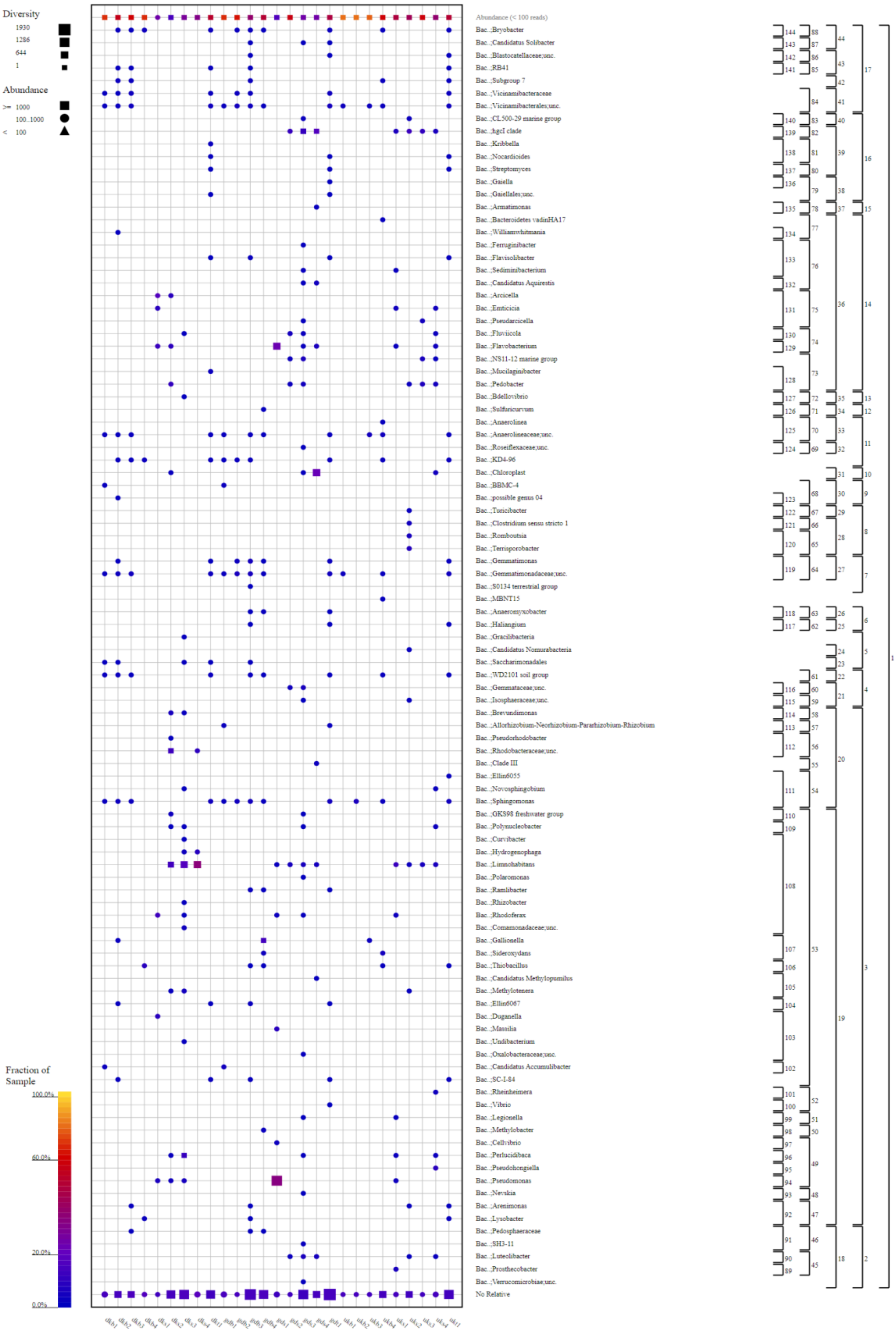

Figure S5. Species-level taxonomic fingerprint of water, soil and sludge samples from rice farming (The figure was created using SILVA NGS 1.4 v138.1).

Table S1. Shannon Index Value of Samples.

Sample Name	*Shannon Index*
**DKB1**	8.57
**DKB2**	8.62
**DKB3**	8.56
**DKB4**	8.20
**DKS1**	7.00
**DKS2**	8.30
**DKS3**	8.42
**DKS4**	7.81
**DKT1**	8.82
**GDB1**	8.41
**GDB2**	8.13
**GDB3**	9.02
**GDB4**	8.65
**GDS1**	8.27
**GDS2**	8.00
**GDS3**	8.65
**GDS4**	8.17
**GDT1**	9.07
**UKB1**	8.23
**UKb2**	7.86
**UKB3**	8.17
**UKB4**	8.70
**UKS1**	8.09
**UKS2**	8.26
**UKS3**	7.55
**UKS4**	8.21
**UKT1**	8.92
